# Colistin Susceptibility Testing by Colistin Broth Disk Elution MIC Method among Carbapenem-resistant Gram-negative Blood Culture Clinical Isolates in a Tertiary Care Setting, East Delhi, India

**DOI:** 10.4314/ejhs.v33i5.4

**Published:** 2023-09

**Authors:** Kirti Nirmal, Narendra Pal Singh, Krishna Sarkar, Nisha Goyal, Seema Gangar, Aditya Nath Dwivedi

**Affiliations:** 1 Department of Microbiology University College of Medical Sciences and Guru Tag Bahadur Hospital Delhi 110095

**Keywords:** Gram-negative, Carbapenem-resistant, Colistin broth disk elution (CBDE)

## Abstract

**Background:**

Antibiotic resistance is a growing concern for bloodstream infections (BSIs), especially with the emergence of multidrug-resistant (MDR) gram-negative bacteria. In this study, we aimed to assess the pattern of colistin susceptibility using the colistin broth disc elution (CBDE) method among carbapenem-resistant gram-negative clinical isolates from blood cultures in a high burden tertiary healthcare setting in East Delhi.

**Methods:**

A total of 106 carbapenem-resistant gram-negative clinical isolates were tested. The most common isolates were Klebsiella pneumoniae, Escherichia coli, Enterobacter species, and Klebsiella oxytoca by CBDE method.

**Result:**

All the carbapenem resistant gram-negative bacterial blood culture isolates showed intermediate colistin susceptibility. This was statistically significant by chi-square test (p<0.5).

**Conclusion:**

This study highlights the need to monitor colistin resistance trends in the face of increasing antimicrobial resistance. Accurate surveillance of emerging colistin resistance is crucial for effective management of BSIs caused by carbapenem-resistant gram-negative bacteria.

## Introduction

Bloodstream infections (BSIs) are defined as the presence of a viable infectious organism in the circulation that produces an inflammatory response manifested by changes in the clinical, biochemical, and hemodynamic parameters (1). In medical practices, bacteremia may range from self-limiting infections to life-threatening septicemia that necessitates immediate and prudent antimicrobial therapy (2). Bacteremia could indeed emerge from invasive devices introducing microorganisms into the bloodstream directly or secondary to infections at other body sites (3).

Over the years, there has been a remarkable transition in the cause of bloodstream infections with the predominance of gram-negative bacteria (2,3). Septicemia is one of the most significant global public health problem worldwide (1,2,3). The challenges associated with BSIs is the continuously evolving antibiotic resistance resulting in poor therapeutic outcomes. Due to the dearth of novel antibiotics for gram-negative pathogens and the severity of illnesses caused by multidrug-resistant (MDR) bacteria, particularly carbapenem-resistant bacteria, older antibiotics are being re-evaluated (4). Resistance to at least one agent in three or more antimicrobial classes is defined as MDR (5). In recent years, colistin, a cationic polypeptide that belongs to the polymyxins class, has gained clinical significance as the last resort antibiotic for the management of multi-drug resistant gram-negative pathogens (6). The interaction between the cationic polypeptides of colistin with the anionic lipopolysaccharide molecules in the outer membrane of gram-negative bacteria lead to displacement of calcium and magnesium causing increased cell permeability and, ultimately, cell death (7). Commercially, two different forms of colistin are available: colistin sulfate, which is primarily used topically, and sodium colistin methanesulfonate, which is administered parenterally. However, both these forms can be administered by the inhalational route (7,8). The major potential toxicities associated with the intravenous administration of colistin are dose-dependent nephrotoxicity and neurotoxicity (9).

Colistin susceptibility testing by disk-diffusion method is unreliable due to poor diffusion of colistin into agar, yielding smaller zones of inhibition (10). The Clinical Laboratory Standard Institute (CLSI) recommends colistin agar test, colistin broth disk elution (CBDE) and colistin broth microdilution minimum inhibitory concentrations (MICs) methods for in-vitro susceptibility testing of colistin in *Enterobacterales* and *Pseudomonas aerugin*osa (11).

The aim of the present study was to observe the pattern of colistin susceptibility by colistin broth disc elution MIC method among the carbapenem- resistant clinical isolates of gram-negative organisms of blood-stream infections in a high burden tertiary health-care setting in East Delhi.

## Material and Methods

This was a cross-sectional study conducted in the Department of Microbiology, UCMS & GTB hospital, Delhi from August to October 2022. A total of 106 carbapenem- resistant gram- negative clinical isolates isolated from blood culture were tested by colistin broth disc elution test. The institutional ethical committee (IEC) has approved this manuscript (N0: GTBHEC/APVL/2022/452-64).

**Blood sample processing**: The blood was manually inoculated into a blood culture bottle and immediately transported to the bacteriology laboratory and incubated at 37°C for overnight incubation in an ambient environment. The samples were manually subcultured onto 5% sheep blood agar and Mac-Conkey agar at 24 hours, 48 hours, and 5^th^ day of incubation. The growth obtained was processed as per the standard protocol (12).

**Antimicrobial susceptibility testing of blood culture isolates**: The antibiotic susceptibility pattern of the isolated organisms was performed by Kirby–Bauer disk diffusion method on Mueller–Hinton agar plates, and the results were recorded as per the CLSI 2022 guidelines. (11) All antibiotics were not tested for all the microorganisms. *Escherichia coli* (ATCC 25922), *Staphylococcus aureus* (ATCC 25923) and *Pseudomonas aeruginosa* (ATCC 27853) control strains were used for the Kirby Bauer disc diffusion method. The carbapenem resistance was detected by the standard phenotypic methods as described in the latest CLSI guidelines.

Colistin broth disk elution test: Colistin was tested by colistin broth disk elution test according to CLSI 2022 guidelines. The principle behind the Colistin broth disc elution test is that antimicrobial discs with known concentrations were eluted in a predetermined volume of broth to produce standard doubling dilutions to measure MICs. Cation-adjusted Mueller-Hinton broth (CA-MHB; Himedia laboratories, Mumbai, India) was used to carry out this method. For each isolate, four tubes containing 10 ml each of CA-MHB were taken. Colistin discs (10 g; BD, Sparks MD) were used to achieve final concentrations of 0 (growth control). 1, 2, and 4 g/ml in the CA-MHB tubes. The tubes were vortexed and left at room temperature for 30 minutes to allow the colistin to adequately elute from the discs. To produce a standardized inoculum, 3-5 colonies from 18–24 hours agar plate were suspended in 4-5 ml of sterile saline. The turbidity was standardized to match that of a McFarland 0.5 standard. To achieve a final inoculum concentration of 7.5 × 10^5^ CFU/ml, a 50 µL aliquot of this standardized inoculum was added to each tube. MIC values were read as the lowest concentration that completely inhibits growth of the test isolate after 16-20 hours incubation at 35°C. Interpretation was done using CLSI breakpoints of 2 g/mL as intermediate and 4 g/mL as resistant (11,13).

**Data management and statistical analysis**: MS Excel spreadsheet program was used to record the data. Frequencies and percentages were used for categorical variables. Group comparisons for categorical data were done using Chi-square test. Statistical significance was kept at p value < 0.05.

## Results

**Figure 1:**
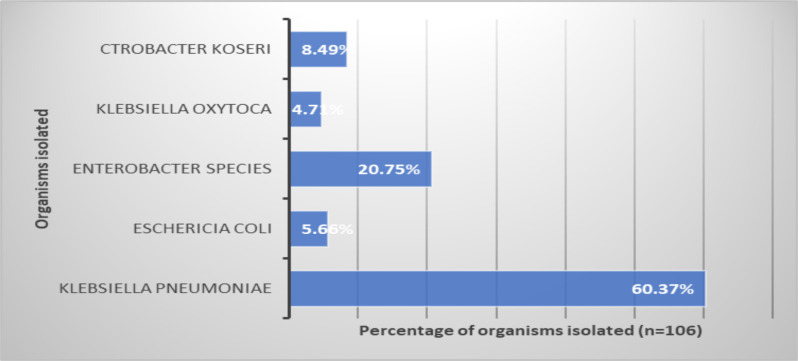
Percentage wise distribution of carbapenem-resistant bacterial blood culture gram-negative clinical isolates (n=106)

In the present study, a total of 106 gram-negative carbapenem-resistant bacterial blood culture isolates were tested for colistin susceptibility testing by colistin broth disk elution test. Overall, the most commonly isolated gram-negative Carbapenem- resistant bacterial blood culture organisms were *Klebsiella pneumoniae*, *Escherichia coli, Enterobacter cloacae* and *Klebsiella oxytoca* (Figure 1).

**Figure 2:**
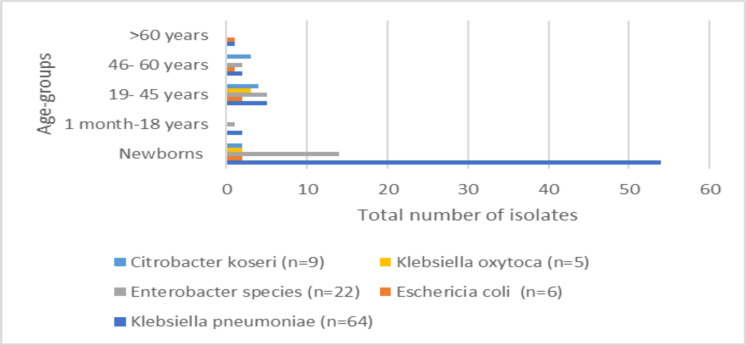
Trends of carbapenem-resistant gram-negative bacterial blood culture clinical isolates among various age-group patients (n=106)

*Klebsiella pneumoniae* accounted for the highest percentage of gram-negative bacterial blood culture isolates (60.37%). The age of the patients ranged from newborns to 60 years, with the male to female ratio of 0.85. This was not statistically significant (paired t test, p>0.67). Trends of gram-negative carbapenem- resistant bacterial blood culture isolates vary with the different age-groups (Figure 2).

**Figure 3:**
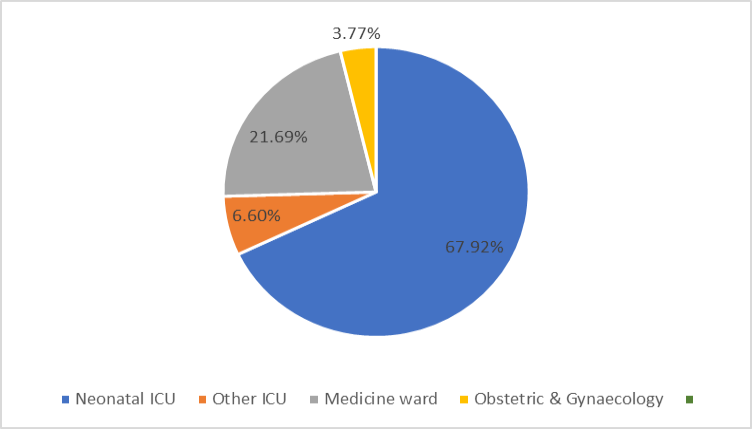
Location wise percentage distribution of carbapenem-resistant gram-negative bacterial blood culture clinical isolates in the study population (n=106)

In newborns, the most commonly isolated MDR bacterial blood culture gram-negative pathogen was *Klebsiella pneumoniae* (72.97%, 54 out of 74), followed by *Enterobacter species* (18.18%, 14 out of 74). In the age category of 46 to 60 years, *Citrobacter koseri* (37.5%, 3 out of 8) and *Klebsiella pneumoniae* (25%, 2 out of 8) were the most frequently isolated carbapenem- resistant gram-negative bacterial blood culture isolates. *Klebsiella pneumoniae* continued to be the most predominantly isolated carbapenem- resistant gram-negative bacterial blood culture isolates in all the other age categories. Maximum carbapenem-resistant gram-negative bacterial blood culture isolates have been isolated from the Neonatal Intensive Care Unit (NICU=67.9%, 72 out of 106) (Figure 3). The trends of antibiotic susceptibility among the carbapenem-resistant gram-negative bacterial blood culture isolates are depicted in Table 1.

**Table 1:** Trends of antibiotic susceptibility among the gram-negative bacterial blood culture clinical isolates (n=106)

	Sensitivity	AMP	GEN	PTZ	CTR	CTX	IMP	MRP	CIP	COT	CAZ	AZT
***Klebsiella pneumoniae* (n=64)**	**Resistant**	**64**	**64**	**64**	**64**	**64**	**64**	**64**	**64**	**44**	**64**	**64**
		**(100%)**	**(100%)**	**(100%)**	**(100%)**	**(100%)**	**(100%)**	**(100%)**	**(100%)**	**(68.75%)**	**(100%)**	**(100%)**
	**Sensitive**	**0**	**0**	**0**	**0**	**0**	**0 (100%)**	**0**	**0**	**20**	**0**	**0**
		**(100%)**	**(100%)**	**(100%)**	**(100%)**	**(100%)**		**(100%)**	**(100%)**	**(31.25%)**	**(100%)**	**(100%)**
***Escherichia coli* (n=6)**	**Resistant**	**6**	**6**	**6**	**6**	**6**	**5**	**6**	**6**	**5**	**6**	**6**
		**(100%)**	**(100%)**	**(100%)**	**(100%)**	**(100%)**	**(83.3%)**	**(100%)**	**(100%)**	**(83.3%)**	**(100%)**	**(100%)**
	**Sensitive**	**0**	**0**	**0**	**0**	**0**	**1**	**0**	**0**	**1**	**0**	**0**
		**(100%)**	**(100%)**	**(100%)**	**(100%)**	**(100%)**	**(16.66%)**	**(100%)**	**(100%)**	**(16.66%)**	**(100%)**	**(100%)**
***Enterobacter species* (n=22)**	**Resistant**	**22**	**22**	**22**	**22**	**22**	**22**	**22**	**22**	**14**	**22**	**22**
		**(100%)**	**(100%)**	**(100%)**	**(100%)**	**(100%)**	**(100%)**	**(100%)**	**(100%)**	**(63.63%)**	**(100%)**	**(100%)**
	**Sensitive**	**0**	**0**	**0**	**0**	**0**	**0 (100%)**	**0**	**0**	**8**	**0**	**0**
		**(100%)**	**(100%)**	**(100%)**	**(100%)**	**(100%)**		**(100%)**	**(100%)**	**(36.36%)**	**(100%)**	**(100%)**
***Klebsiella oxytoca* (n**=5)	**Resistant**	**5**	**5**	**5**	**5**	**5**	**5 (100%)**	**5**	**5**	**4 (80%)**	**5**	**5**
		**(100%**	**(100%**	**(100%**	**(100%**	**(100%**		**(100%**	**(100%**		**(100%**	**(100%**
	**Sensitive**	**0**	**0**	**0**	**0**	**0**	**1 (20%)**	**0**	**0**	**1 (20%)**	**0**	**0**
		**(100%)**	**(100%)**	**(100%)**	**(100%)**	**(100%)**		**(100%)**	**(100%)**		**(100%)**	**(100%)**
***Citrobacter koseri* (n=9)**	**Resistant**	**9**	**9**	**9**	**9**	**9**	**8**	**9**	**9**	**8**	**9**	**9**
		**(100%)**	**(100%)**	**(100%)**	**(100%)**	**(100%)**	**(88.8%)**	**(100%)**	**(100%)**	**(88.8%)**	**(100%)**	**(100%)**
	**Sensitive**	**0**	**0**	**0**	**0**	**0**	**1**	**0**	**0**	**1**	**0**	**0**
		**(100%)**	**(100%)**	**(100%)**	**(100%)**	**(100%)**	**(11.11%)**	**(100%)**	**(100%)**	**(11.1%)**	**(100%)**	**(100%)**

AMP = ampicillin(10ug), GEN = gentamycin(10ug), PTZ = piperacillin + tazobactam(100/10ug), CTR = ceftriaxone(30ug), CTX = cefotaxime(30ug), IPM = imipenem(10ug), MRP = meropenem(10ug), CIP = ciprofloxacin(15ug), COT = cotrimoxazole (trimethoprim + sulfamethoxazole) (1.25/23.75ug), CAZ = ceftazidime(30ug), AZT = aztreonam(30ug).

All the carbapenem resistant gram-negative bacterial blood culture isolates have shown intermediate colistin susceptibility (Colistin MIC ≤2µg/ml). This was statistically significant by chi-square test (p<0.5) (Table 2).

**Table 2:** Trends of colistin susceptibility pattern among the carbapenem-resistant gram-negative bacterial blood culture clinical isolates by colistin broth disk elution test (n=106)

Colistin category	*Klebsiella pneumoniae (n=64)*	*Escherichia coli (n=6)*	*Enterobacter species (n=22)*	*Klebsiella oxytoca (n=5)*	*Citrobacter koseri (n=9)*
Colistin MIC ≤2µg/ml (Intermediate)	64 (100%)	6 (100%)	22 (100%)	5 (100%)	9 (100%)
Colistin MIC ≥4µg/ml (Resistant)	0	0	0	0	0

## Discussion

No microbiological test, biomarker, or clinical score demonstrates optimal predictive value for the diagnosis of bloodstream infections and sepsis, which presents a challenge for the physicians (14). The majority of cases of the community and hospital-acquired sepsis and septic shock are caused by bloodstream infections (15). Carbapenem-resistant pathogens causing bloodstream infections can have substantial fatality rates, particularly when the pathogens are resistant to carbapenems as well as colistin. Since no novel medications are in development, understanding colistin resistance is crucial. Except for bacteria from the genera *Proteus, Providencia, Morganella, Serratia, Edwardsiella*, and *Burkholderia*, gram-negative bacteria are known to be susceptible to colistin (16). Our study documented *Klebsiella pneumoniae* as the most commonly isolated multi-drug resistant gram-negative blood culture pathogen. A study conducted by *Orsini et al* on microbiological profile of organisms causing bloodstream infection in critically ill patients also reported *Klebsiella pneumonia* to be most commonly isolated multi-drug resistant gram-negative bacteria, similar to our findings (17). Similar results have also been documented in another study by *Yusuf I. et al* in West Nigeria (18). The recent emergence of hypervirulent strains of *Klebsiella pneumoniae*, involved in mild to severe illnesses raises concerns about a possible threat (19).

One-third of all gram-negative infections are caused by nosocomial *Klebsiella pneumoniae*, and it is also associated with increased carbapenem resistance globally (20). Another study evaluating the trends of bloodstream infections among pediatric and adult patients also found *Klebsiella species* to be the most commonly isolated gram-negative pathogen (2). *Klebsiella pneumoniae* is associated with high colistin resistance. The high colistin usage rate in clinical settings to treat carbapenem-resistant *Klebsiella pneumoniae* during the pandemic has probably contributed to increased colistin resistance seen in bloodstream *Klebsiella pneumoniae* isolates (20).

In this study, higher resistance was observed among the ampicillin, broader spectrum cephalosporins, aminoglycosides, monobactams, fluoroquinolones, piperacillin-tazobactam and meropenem. While cotrimoxazole resistance ranged from 63.63% to 88.8%, imipenem resistance ranged from 83.3% to 100%. A study conducted in Nepal also had similar findings (2). Antibiotics being indiscriminately administered to both inpatients and outpatients might be the cause of the high level of resistance seen in developing nations.

In the present study, all the isolates have shown intermediate colistin susceptibility (Colistin MIC ≤ 2µg/ml). Mostly chromosomal mutations in Two Component Systems (TCS) PhoPQ and PmrAB and plasmid-borne (*mcr* and its variants) genes are responsible for Colistin resistance in Gram negative bacteria (21). Colistin resistance in *Klebsiella pneumoniae* is mediated by a variety of mechanisms, including changes in lipid A and lipopolysaccharides, efflux pumps, outer-membrane alterations, and changes in the capsular polysaccharide and type (19). According to a recent meta-analysis report on the global prevalence of colistin resistance in *Klebsiella pneumoniae* from bloodstream infections, Thailand had the highest percentage of colistin resistance (19.2%), while South Korea had the lowest rate (0.8%). The same study reported 7.6% pooled colistin resistance in bloodstream infection caused by *Klebsiella pneumoniae* from India (20,21). In another study on the prevalence of colistin resistance among MDR isolates of ICU patients in India found 23% colistin resistance in MDR organisms isolated from blood culture (22).

This study provides valuable insights into the colistin susceptibility pattern among carbapenem-resistant blood culture isolates in a tertiary healthcare setting. Given the alarming rise of antimicrobial resistance, monitoring the trends, patterns, and prevalence of colistin resistance becomes imperative. Such surveillance will provide reliable information and enable effective management of emerging colistin resistance.

Additionally, it is crucial to implement colistin stewardship programs to prevent the indiscriminate use of this antibiotic. Judicious and responsible use of colistin is necessary to preserve its effectiveness and minimize the development of further resistance. Hence, we can enhance patient outcomes and preserve the effectiveness of our limited antibiotic arsenal in the face of antimicrobial resistance.
